# Orbital Metastasis from Rectal Adenocarcinoma- Report of a Rare Case

**Published:** 2017-01-02

**Authors:** Subrata Pal, Kingshuk Bose, Abhishek Sharma, Mrinal Sikdar

**Affiliations:** 1 *Dept. of Pathology, College of Medicine and SagoreDutta Hospital, India*; 2 *Dept. of Pathology, BankuraSammilani Medical College, India*; 3 *Dept of Pathology, Kolkata National Medical College, India*

**Keywords:** Orbital Metastasis, Adenocarcinoma, Rectum

## Abstract

Colorectal carcinoma is a common malignancy in India as well as in world. Inspite of its high metastasizing ability to various organs and lymph node, orbital metastasis is exceptional. Very few cases have been reported in the world literature. We report orbital metastasis in a case of moderately differentiated rectal adenocarcinoma in a 58-year male patient from India in 2015. We want to focus on the rare metastatic pathway of rectal adenocarcinoma and early diagnosis of the orbital metastasis, which can help in application of therapy to save the eyesight.

## Introduction

Colorectal carcinoma is a common malignancy in India as well as in the world (1). About 20% of colorectal carcinomas present with the distant metastasis at the time of diagnosis. Other 30% colorectal carcinomas develop metastasis during the course of the disease (1). Most of the secondary orbital tumors arise from the adjacent organs (2). Secondary orbital malignancies from distant organs are uncommon and usually arise from breast carcinoma, lung malignancies, melanoma and prostatic carcinomas. Orbital metastasis from rectal adenocarcinoma is very rare occurrence and only a few cases have been reported until now (3, 4). 

Here we report unilateral orbital and brain metastases in a case of moderately differentiated adenocarcinoma of rectum in an elderly male from tribal region of eastern India.

## Case report

A A 62-year male patient from tribal region in West Bengal, India was attended to Surgery Out Patient Department in 2015 with complaints of irregular bowel habit and occasional rectal bleeding for last two months. He had average built and had a history of weight loss. Rectal examination revealed an elevated irregular mass at posterior part of rectum. Ultrasound and CT scan of abdomen and pelvis were done and revealed a circumscribed solid hypodense mass at rectum without any adhesion or extension to other pelvic organs. Ultrasound showed multiple nodular hypodense spaces occupying lesion of the liver, suggestive of metastases.

**Fig. 1:**
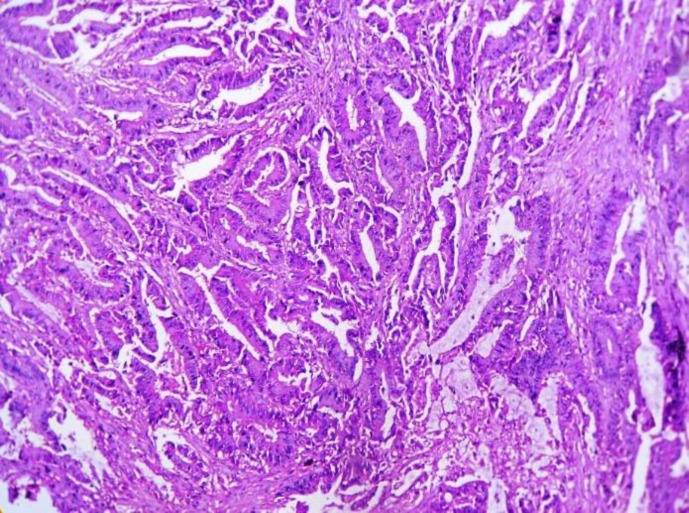
Photomicrograph shows irregular distorted glands lined by highly dysplastic glandular cells with severe atypia and mitosis (H & E stain, 10X view

He was subsequently undergone colonoscopy and biopsy from the lesion after taking informed consent. On colonoscopy, there was an elevated irregular rectal mass 3.5 cm above the anal verge. Histopathology showed irregular distorted glands lined by highly dysplastic glandular cells with severe atypia and mitosis. A biopsy was reported as moderately differentiated adenocarcinoma of rectum (Fig. 1). As it was small endoscopic biopsy, extension to layers could not be assessed. Immunostain was positive for CK20 and negative for CK 7. 

**Fig. 2:**
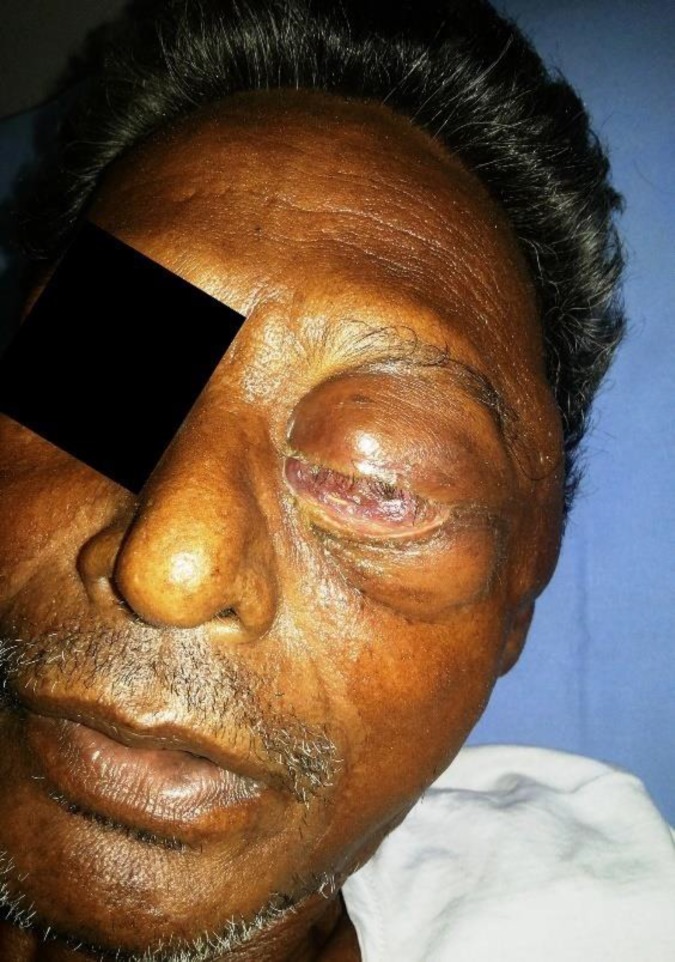
Gross image of the left eye of the patient showing enlarged eyeball with conjunctival chemosis

**Fig. 3:**
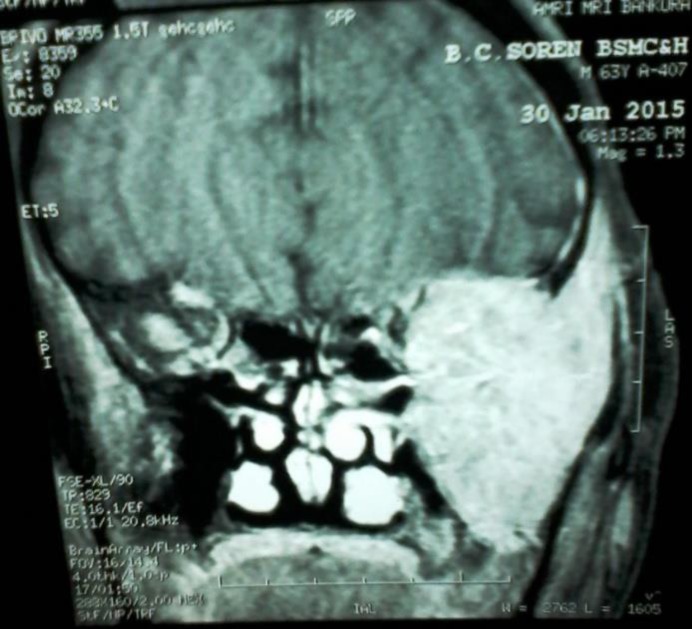
Photomicrograph of MRI brain and orbit showed a soft tissue swelling in left infratemporal fossa measuring 5.2x4 cm extending medially to infiltrate the left orbit and its bony lateral wall

Other biochemical and hematological tests revealed only significant anemia (hemoglobin-7.8gm/dl). Chest X-ray did not reveal any sign of metastatic lesion. He was planned for surgery and further management. In the meanwhile he developed pain, gradual dimness of vision and swelling of left eyeball (proptosis). He was referred for ophthalmologic examination.On ophthalmologic examination, he had proptosis of left eye, restriction of lateral and upper gazes (Fig.2). He had visual acuity of 20/40 in right eye and there was no perception of light in left eye. On fundoscopic examination, he had subretinal collection of fluid suggesting retinal detachment but no any choroidal lesion was found. He underwent MRI of brain and orbit revealed a soft tissue swelling inthe left infratemporal fossa measuring 5.2x4 cm. It was extended medially and infiltrated the left orbit and its bony lateral wall besides the lateral rectus muscle (Fig. 3). Left optic nerve was abutted and buckled and post-superior wall of the left maxillary sinus is abutted laterally. The mass was hypodense on T1W1 and hyperintense on T2W1 showing marked enhancement. MRI diagnosis was metastatic tumoral infiltration of the left orbit with left infratemporal fossa extension.

He was further treated with adjuvant whole brain radiotherapy (30 Gy) and chemotherapy (5FU). After second cycle, he developed severe thrombocytopenia, febrile neutropenia and died after four daysdespite the best supportive care

## Discussion

Metastatic orbital tumors are very rare accounting 2%-7% of orbital tumors in different series (2,5). Most of the metastatic orbital tumors arise from adjacent organs (2). Among the distant primary sites common are breast, prostate, and lung (1). Only 4% of orbital metastases originate from gastrointestinal primary (1). Incidence of ocular metastasis is much higher than orbital metastasis and metastatic tumors are the most common causes of orbital malignancies (1,2). 

Colorectal carcinoma is very common malignancy in India as well as in South East Asia. About 1/5^th^ of the cases have distant metastasis at diagnosis and other 30% develop metastasis during disease progression (1). Common sites of secondary involvement from colorectal carcinoma are “liver (77%), peritoneum (25%) and lung (22%)” (1). Though incidence and prevalence of rectal carcinoma is high throughout world, ocular and orbital metastases from colo-rectal carcinoma are extremely rare incident (3). Symptoms of orbital metastasis are diplopia, pain and gradual dimness of vision. Clinical signs are proptosis, alteration of eye motility, palpebral ptosis, displacement of eye globe, conjunctival chemosis, and enophthalmos (2). In our case, proptosis and diplopia were the early symptoms. 

Most of the previous cases of orbital metastasis from rectal carcinomas have been developed during disease progression except in one case where primary malignancy was diagnosed after the onset of visual disturbance (1,6). In our case, also rectal carcinoma was diagnosed two months before the ocular symptoms.

Theoretically, orbital metastasis from rectal adenocarcinoma occurs through two possible pathways. It can spread “through middle or inferior haemorrhoidal veins and followed by inferior vena cava, pulmonary circulation, carotid arteries and ophthalmic arteries” (7). Another way of seeding is via Batson’s venous plexus to cranial venous sinus followed by ophthalmic vein (3, 7). It can spread to lungs through inferior vena cava. When rectal adenocarcinoma spreads via the path of Batson’s venous plexus, vertebral metastasis is common (3).However, most of the rectal adenocarcinomas spread via portal venous pathway, leading to hepatic metastasis. It also provides a barrier to spread via systemic circulation. This is the probable reason behind the rare incidence of orbital metastasis in rectal carcinoma. 

Most of the previous cases of ocular metastasis from rectal adenocarcinoma, also, exhibit other systemic metastases due to long pathway of systemic circulation (1). In this case, the patient had liver metastasis in the absence of lung metastasis. MRI of orbit and brain is most useful imaging to assess the metastatic tumor, and its extension and intracranial spread. Most similar previous cases were treated with palliative chemotherapy, locoregional radiation and intravenous bevacizumab (1,8,9). Prognosis of orbital metastasis depends on location, type, and differentiation of primary tumor and the time of metastasis since diagnosis of primary tumor (2). Overall, prognosis is poor and average survival is only a few months (8,9). This patient died due to chemotherapy-induced agranulocytosis and sepsis.

## Conclusion

Orbital metastasis from rectal adenocarcinoma is very rare. We want to focus on the rare metastatic pathway of rectal adenocarcinoma because early diagnosis can help in application of therapy to save the eyesight. 

## Conflict of Interests

The authors declare that there is no Conflict of Interests. 
